# Euphorbia factor L3 ameliorates rheumatoid arthritis by suppressing the inflammatory response by targeting Rac family small GTPase 1

**DOI:** 10.1080/21655979.2022.2066761

**Published:** 2022-04-27

**Authors:** Haojun Shi, Shufeng Li, Yun Geng, Huancai Fan, Ruojia Zhang, Yuang Zhang, Jihong Pan, Guanhua Song, Luna Ge, Tianhua Xie, Lin Wang

**Affiliations:** a The Second Clinical Medical College, Henan University of Chinese Medicine, Zhengzhou, Henan, China; b Department of Orthopedic Surgery, The First Affiliated Hospital of Shandong First Medical University, Jinan, Guangdong, China; c Shandong First Medical University & Shandong Academy of Medical Sciences, Jinan, Shandong, China; d Biomedical Sciences College & Shandong Medicinal Biotechnology Center, NHC Key Laboratory of biotechnology drugs (Shandong Academy of Medical Sciences), Key Laboratory for Rare & Uncommon Diseases of Shandong Province, Shandong First Medical University & Shandong Academy of Medical Sciences, Jinan, Shandong, China; e Institute of Basic Medicine, Shandong First Medical University & Shandong Academy of Medical Sciences, Jinan, Shandong, China; f Department of Rheumatology and Immunology, Shandong Provincial Hospital Affiliated to Shandong First Medical University (Shandong Provincial Hospital), Jinan, Shandong, China

**Keywords:** Euphorbia factor L3, inflammation, NF-κB, rheumatoid arthritis, Rac1

## Abstract

Euphorbia factor L3 (EFL3) is extracted from *Euphorbia lathyris* and is known for its anti-inflammatory properties. This study focused on the potential anti-inflammatory and therapeutic effects of EFL3 on rheumatoid arthritis (RA) using fibroblast-like synoviocytes (FLSs) and arthritis animal models. Functional analysis showed that EFL3 could ameliorate the inflammatory phenotype of FLSs derived from RA patients, as evidenced by the decreases in cell viability, migration, invasion and cytokine production. Luciferase activity, Western blotting and immunofluorescence assays demonstrated that EFL3 inhibited the nuclear translocation of the p65 subunit and the subsequent activation of the nuclear factor kappa-Β (NF-κB) pathway. Furthermore, the therapeutic effects of EFL3 against arthritic progression were evidenced by decreases in joint swelling, arthritis scores, inflammatory factor production, synovial hyperplasia, and bone destruction in collagen-induced arthritis (CIA) and tumor necrosis factor-α (TNF-α) transgenic (TNF-tg) mouse models. Molecular analysis identified Rac family small GTPase 1 (Rac1) as the potential target that was required for EFL3-mediated suppression of the inflammatory RA FLS phenotype. In summary, this study uncovered the therapeutic potential of EFL3 in RA, which suggests its future clinical use.

## GRAPHICAL ABSTRACT

**Figure:**
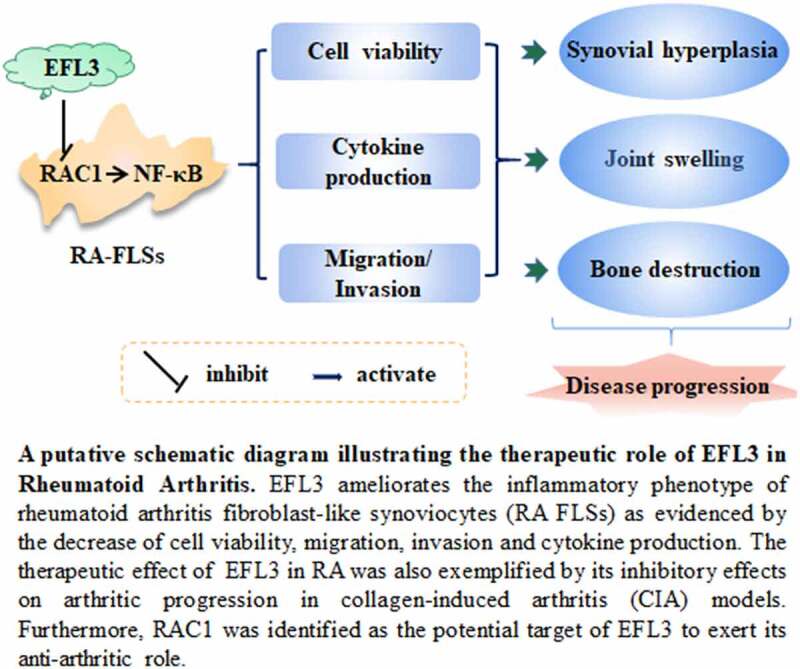

## Highlights

- EFL3 suppresses the inflammatory manifestations of RA FLSs.
- EFL3 inactivated the NF-ĸB signaling pathway in RA FLSs.
- Rac1 serves as the potential target by which EFL3 exerts its anti-inflammatory effects.

## Introduction

Rheumatoid arthritis (RA) is a chronic inflammatory disease characterized by high synovial proliferation and inflammation that leads to severe joint destruction [1]. Although advances in therapeutic strategies have significantly improved clinical outcomes, a notable percentage of RA patients cannot achieve clinical remission and become resistant to existing therapeutic strategies [2]. It is therefore urgent to identify novel drugs with the potential for RA treatment.

Recently, small molecule inhibitors targeting classic proinflammatory signaling pathways have served as promising treatments for RA patients [3]. Nuclear factor kappa-Β (NF-κB) is closely related to RA progression, as it can promote the production of inflammatory cytokines such as interleukin-1 (IL-1), tumor necrosis factor-α (TNF-α) and interleukin-6 (IL-6) in fibroblast-like synoviocytes (FLSs) and macrophages [4]. Importantly, these cytokines all contribute to bone erosion and disease progression [4]. Therefore, identifying an effective inhibitor of NF-κB signaling could provide a novel treatment strategy for RA.

Euphorbia factor L3 (EFL3) is one of the active components of *Euphorbia lathyris*, a traditional Chinese herbal medicine that has been used to treat terminal schistosomiasis, amenorrhea, ascites, constipation, and snakebites [5]. Here, our study showed that EFL3 could inactivate the NF-κB signaling pathway in RA FLSs. Previous studies have shown that EFL3 can inhibit the expression of proinflammatory factors, such as inducible nitric oxide synthase (iNOS), and decrease the production of nitric oxide (NO) [6]. Importantly, iNOS and NO are also downstream targets of NF-κB [7,8] and are closely related to the inflammatory responses triggered by IL-1, TNF-α and IL-6 [9–12]. These findings indicate the potential anti-inflammatory role of EFL3 in RA.

In this study, we hypothesized that EFL3 prevented the inflammatory progression of RA. To confirm our hypothesis, this study examined the effects of EFL3 on the viability, migration, invasion, and cytokine production of FLSs derived from RA patients. In vivo experiments using collagen-induced arthritis (CIA) and TNF-α transgenic (TNF-tg) mouse models were also performed to investigate the therapeutic potential of EFL3 and its underlying mechanism.

## Materials and methods

### Cell culture and reagents

Synovial tissues from RA patients who underwent synovectomy or joint replacement surgery at Shandong Provincial Hospital (Jinan, China) were collected, and RA FLS isolation and culture were performed as described previously [13]. Tohoku Hospital Pediatrics-1 (THP-1) -Lucia™ NF-κB cells were purchased from Invitrogen (Carlsbad, CA, USA). This study was approved by the Medical Ethics Committee of the Institutional Review Board of Biomedical Sciences College, and informed consent was obtained from all patients (SMBA20LL017).

### Small interfering RNA (siRNA) transfection and quantitative real-time PCR (qRT–PCR)

RA FLSs (2 × 10^5^ cells in 100-mm dishes or 8.5 × 10^4^ cells in 6-well plates) were plated, grown to 75%confluence and were transiently transfected with the indicated siRNA. siRNA against RAC1 and the negative control were designed and purchased from Ruibo (Guangzhou Development Zone, Guangzhou, China) and transfected into RA FLSs using transfection reagents (101,000,028, Polyplus-transfection, Strasbourg, France) according to the manufacturer’s instructions. The sequences of the siRNA targeting human RAC1 (siRAC1) were as follows: sense strand: 5'-GGGAUGAUAAAGACACGAUTT-3' and antisense strand 5'-AUCGUGUC UUUAUCAUCCCTT-3'. The sequences of the negative control siRNA (siNC) were as follows: sense strand: 5'-UUCUCCGAACGUGUCACG-3' and antisense strand: 5'-ACGUGACACG UUCGGAGAATT-3'. After total RNA was extracted and reverse transcribed, qRT–PCR was performed using the 2-ΔCycle threshold (2-ΔCT) method [14]. All primers (Supplementary Table 1) were synthesized by Beijing Genomics Institute (BGI, Beijing, China).

### Cell Counting Kit-8 assay (CCK-8), luciferase activity assay and enzyme-linked immunosorbent assay (ELISA)

To perform the CCK-8 assay, 2 × 10^3^ RA FLSs were incubated with different doses of EFL3 (S9279, Selleck Chemicals, Houston, TX, USA) after the cells had adhered. CCK-8 solution (B34304, Bimake, Houston, USA) was added, and the absorbance at 450 nm was measured (SpectraMax ID3, Molecular Devices, Shanghai, China). A total of 2 × 10^4^ THP-1-Lucia™ NF-κB cells were incubated with lipopolysaccharide (L5293, LPS fromEscherichia coli J5; Sigma, St Louis, MO, USA 1 µg/ml) or EFL3 (20 µM) for 24 h. Then, 10 μL of the cell suspension was transferred into a 96-well opaque plate, and 50 μL of Quanti-Luc™ solution was added. Luciferase activity was measured immediately using a microplate reader (SpectraMax ID3, Molecular Devices, Shanghai, China). Interleukin-1α (IL-1α), IL-6, and interleukin-8 (IL-8) in the cells supernatant or mice serum were measured using commercial ELISA kits (70-EK101A-96, Lianke, Hangzhou, China).

### Cell migration and invasion assays

RA FLSs were pretreated with TNF-α or interleukin-1β (IL-1β) (300–01a-10, Peprotech, New Jersey, USA, 10 ng/ml) and subsequently challenged with EFL3 for 24 h. Migration and invasion assays were performed using Transwell chambers with 8-μm pores (3384, Corning, Tewksbury, MA, USA) without or with Matrigel (10,165,921,001, Merck, USA), respectively [13]. For the invasion assay, the membrane of the upper chamber was precoated with Matrigel (Corning, Tewksbury, MA, USA), and the cells were seeded as in the migration assay and allowed to invade for 24 h. The data are shown as the mean number of migrated or invaded cells in six randomly chosen fields.

### Network pharmacology and molecular docking analysis

Network pharmacology was used to predict the targets of EFL3 as previously described [15]. TargetNet (http://targetnet.scbdd.com/calcnet/index), STITCH (http://stitch.embl.de), and PharmMapper (http://lilabecust.cn/pharmmapper) were used, and duplicates derived from combining the targets of EFL3 predicted from these methods were deleted. Then, the National Center for Biotechnology Information (NCBI) gene database, Gene Ontology (GO), Kyoto Encyclopedia of Genes and Genomes (KEGG) and STRING were used to search for the potential targets of EFL3 in RA. The Yinfo Cloud Computing Platform was used for molecular docking analysis (https://cloud.yinfotek.com).

### Drug affinity responsive target stability (DARTS) assay and cellular thermal shift assay (CETSA)

DARTS and CETSA were performed as described previously [16]. After the cells were fully lysed, protein lysate and EFL3 were gently mixed and incubated to allow for sufficient binding and digestion by pronase (P8360, Solarbio, Beijing, China). Then, the protein samples were mixed with 5× protein loading buffer and subjected to Western blot analysis. For CETSA, cells in 10-cm plates that had reached 80%-90% confluence were treated with EFL3 or dimethyl sulfoxide (DMSO) (D8371, Solarbio, Beijing, China) for 1 h. Then, the samples underwent trypsinization, heat treatment at the indicated temperature, centrifugation and Western blotting.

### Western blotting

Cells were collected and lysed with radioimmunoprecipitation assay (RIPA) buffer (P0013B, Beyotime Biotechnology, Shanghai, China) supplemented with aprotease inhibitor cocktail (P1006, Beyotime Biotechnology, Shanghai, China). Total protein was subjected to Western blotting as described previously [17]. Nuclear proteins were extracted using Nuclear and Cytoplasmic Extraction Reagent kits (78,835, Thermo Scientific, Waltham, MA, USA). The primary antibodies used in our study were as follows: p65 (ab16502 1:1000), p-p65 (ab76302, 1:1000), Rac1 (ab155938, 1:1000), GAPDH (ab9485, 1:1000) and histone H3 (ab1791, 1:1000). The secondary antibodies used wereHRP-coupled anti-mouse IgG and anti-rabbit IgG (GEHealthcare). Antibody binding was detected by enhancedchemiluminescence with hyperfilm ECL or an RGB 600Imager (GE Healthcare).

### Plasmid transfection

Full-length human Rac1 cDNA was amplified by reverse transcription PCR and cloned into pCR8/GW/TOPO (K280020, Invitrogen, Waltham, USA) as described previously [18]. The amino acid residue Cys18 is located in the guanosine triphosphatase (GTPase) domain of Rac1 and is pivotal for catalysis. The C18A mutant of Rac1 was constructed with a QuikChange site-directed mutagenesis kit (200,523, Agilent, CO, USA) [19].

### Arthritis mouse models and evaluation

CIA models were established as previously described [13]. Briefly, bovine collagen type II (CCII) (2 mg/ml) was mixed with complete Freund’s adjuvant (CFA) (2 mg/ml *Mycobacterium tuberculosis*; Chondrex, Inc.) and injected intradermally on day 0 at the base of the tail with 100 μL of the emulsion into 8- to 11-week-old DBA/1 J mice. On day 21,the mice received an intraperitoneal booster injection of 100 μg of CCII in incomplete Freund’s adjuvant (IFA). Toinvestigate the treatment efficacy of EFL3 at diseaseonset, on day 21 postimmunization, the mice were divided into two groups at random, which were intraperitoneally injected with solvent control or EFL3 (10 mg/kg) from days 22 to 42 (the primary immunization day was day 0). Healthy DBA mice were administered the solvent control as the normal negative control. All model mice were housed under specific pathogen-free conditions, and these studies were approved by the Institutional Animal Care and Use Committee of Shandong First Medical University (SMBC21LL006). TNF-Tg mice (3647 line) with a C57BL/6 background were obtained from Dr. G. Kollias (Institute of Immunology, Alexander Fleming Biomedical Sciences Research Center, Vari, Greece). Sixteen-week-old male heterozygous TNF-Tg mice were subjected to EFL3 treatment. The protocols for the drug intervention were the same as those for CIA mice. The arthritis score was assessed continuously in both CIA and TNF-tg mice as described previously [20]. Mouse joints and human synovial tissues were subjected to H&E staining for histological assessments. Micro computed tomography (Micro-CT) (Quantum GX, Perkin Elmer, USA) was conducted to visualize the microstructure of the joint as described in our previous study [16].

### GTPase activity assay

The effect of EFL3 on RAC1 GTPase activity was determined using a GTPase-Glo™ Assay (v7681, Promega, Wisconsin, USA). Specifically, 25 μl of guanosine triphosphate (GTP) diluted with kinase reaction buffer, 25 μl of recombinant human Rac1 protein (ab268912, Abcam, UK) pretreated with or without EFL3, and 50 μl of detection reagent were added to a 96-well plate. After being incubated at room temperature for 20 min, chemiluminescence signals were measured to generate a GTP standard curve. GTPase activity was calculated based on the standard curve.

### Statistical analysis

GraphPad Prism 9 software (GraphPad Software, Inc., San Diego, CA) was used for all statistical analyses. All data are expressed as the mean ± standard deviation (SD). One-way and two-way analysis of variance was used for comparisons between multiple groups, and the least significant difference t test (LSD-t test) was used for further pairwise comparisons. The Levene test was used for homogeneity of variance analysis, and factors between groups were analyzed interactively. P < 0.05 was considered statistically significant.

## Results

### EFL3 inhibits the activity of the NF-κB signaling pathway

**Figure 1.**
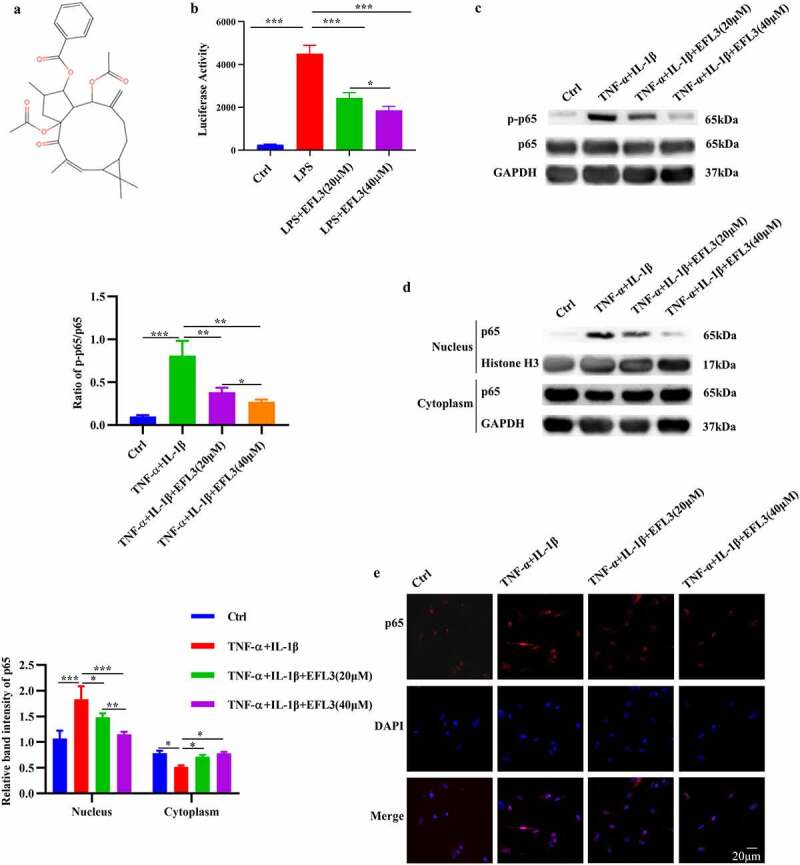
EFL3 inactivates the NF-κB signaling pathway. (a) Structural formula of EFL3. (b) Luciferase activity was analyzed when THP-1-Lucia™ NF-κB cells were treated with LPS (1 µg/ml) with or without EFL3 (20, 40 µM) for 24 h. (c) The phosphorylation of p65 was evaluated by Western blotting. The band intensities were quantified by ImageJ software. (d) Protein extracts from the nucleus and cytoplasm were subjected to Western blotting to measure p65 protein expression. The band intensities were quantified using ImageJ software. (e) RA FLSs were pretreated with EFL3 (20 µM, 40 µM) for 1 h and subsequently treated with TNF-α (10 ng/ml) or IL-1β (10 ng/ml) for another 30 minutes. The cells were fixed and subjected to immunofluorescence staining using a specific antibody against p65. The data are shown as the mean ± SD (*n*= 3). * *p* < 0.05, ** *p* < 0.01, *** *p* < 0.001.

NF-κB is one of the key inflammatory pathways in RA, and it contributes to the excessive expression of inflammatory cytokines such as TNF-α, IL-6, and matrix metalloproteinases (MMPs), which are linked to bone erosion and disease progression [4,21]. Thus, we evaluated the effect of EFL3 (Figure 1(a)) on the activity of the NF-κB pathway, and human THP-1 monocytes stably integrated with an NF-κB-inducible luciferase (Luc) reporter construct were used. As shown in Figure 1(b), EFL3 markedly attenuated the dose-dependent increase in luciferase activity induced by LPS.

Without inflammatory stimulation, p65 localized almost entirely in the cytoplasm in macrophages, while it was concentrated in the nucleus after TNF-α stimulation, indicating its activation (Figure 1(c)). This nuclear translocation was inhibited to a great degree by culturing the macrophages with EFL3 (Figure 1(c)). Additionally, the phosphorylation level of p65 (p-p65), which indicates its activity, was also measured. The Western blot results showed a noticeable increase in p65 phosphorylation following TNF-α exposure. In contrast, EFL3 efficiently inactivated the NF-κB signaling pathway and dose-dependently reduced p65 phosphorylation (Figure 1(d)). To further confirm the effect of EFL3 on p65 translocation, immunofluorescent staining was performed on RA FLSs. As shown in Figure 1(e), the TNF-α-induced increase in p65 protein in the nuclear compartment was significantly attenuated by EFL3. Together, these results indicate that EFL3 reduces the phosphorylation, nuclear translocation and consequent activity of p65.

### EFL3 attenuates the inflammatory phenotype of RA FLSs

**Figure 2.**
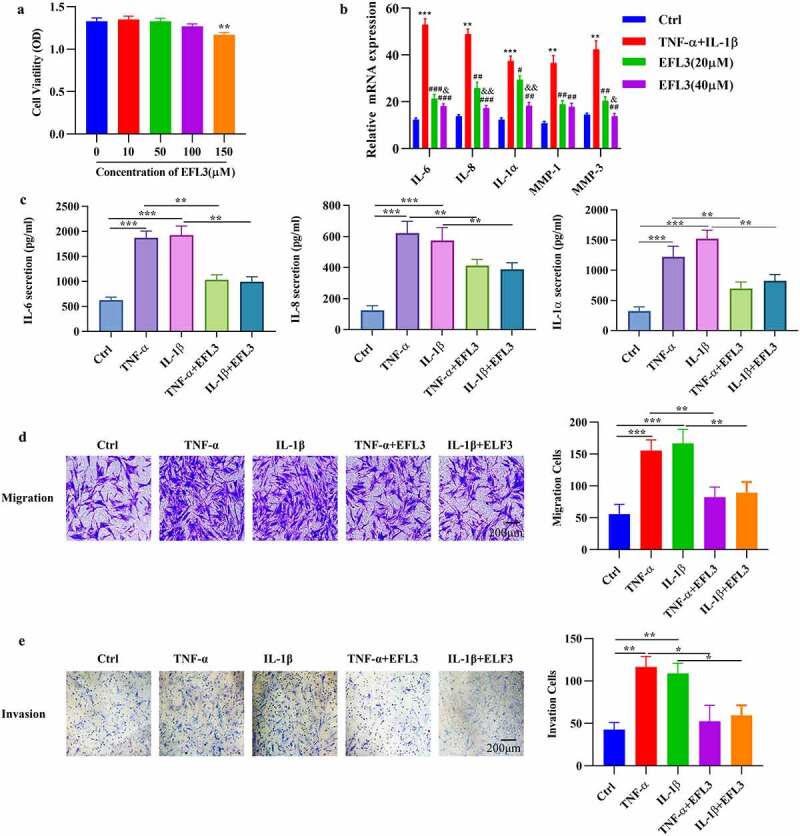
EFL3 inhibits the inflammatory phenotype of RA FLSs. (a) RA FLSs were treated with EFL3 at the indicated concentrations for 72 h, and cell viability was measured by CCK-8 assays. (b) RA FLSs were pretreated with EFL3 (20 μM, 40 μM) for 6 h and subsequently treated with TNF-α (10 ng/ml) or IL-1β (10 ng/ml) for an additional 48 h. The transcriptional levels of IL-6, IL-8 and IL-1α were determined by RT–qPCR. ** *p* < 0.01, *** *p* < 0.001 compared to Ctrl; ^##^ *p* < 0.01, ^###^ *p* < 0.001 compared to TNF-α+ IL-1β; ^&^*p* < 0.01, ^&&^*p* < 0.001 compared to TNF-α+ IL-1β+EFL3 (20 μM). (c) RA FLSs were pretreated with EFL3 (20 μM) for 6 h and subsequently treated with TNF-α (10 ng/ml) or IL-1β (10 ng/ml) for an additional 48 h. The production of IL-6, TNF-α, and IL-1α in the supernatant was measured by ELISA. The effect of EFL3 on the migration (d) and invasion (e) of RA FLSs was measured by transwell assays without or with matrigel. The data are presented as the mean ± SD (*n* = 6). * *p* < 0.05, ** *p* < 0.01, *** *p* < 0.001.

In the inflamed joints of RA patients, FLSs proliferate abnormally and produce inflammatory cytokines and chemokines [22]. Additionally, activated FLSs gain an aggressive phenotype and contribute to the transformation of invasive hyperplastic tissue [2,23]. We therefore evaluated the effect of EFL3 on the proliferation of RA FLSs by CCK-8 assays, and the data showed that treatment with EFL3 did not obviously disturb the viability of RA FLSs (Figure 2(a)). Then, the effect of EFL3 on the inflammatory response of RA FLSs exposed to TNF-α or IL-1β was evaluated by analyzing the production of inflammatory cytokines. As shown in Figure 2(b,c), EFL3 treatment inhibited the expression and secretion of IL-6, IL-8 and IL-1α in a dose-dependent manner. Next, RA FLSs were pretreated with EFL3 for 6 h and then treated with TNF-α or IL-1β for 48 h. Transwell assays with or without Matrigel showed that EFL3 suppressed the migration and invasion of FLSs (Figure 2(d,e)).

### EFL3 prevents inflammation and bone erosion in arthritis models

**Figure 3.**
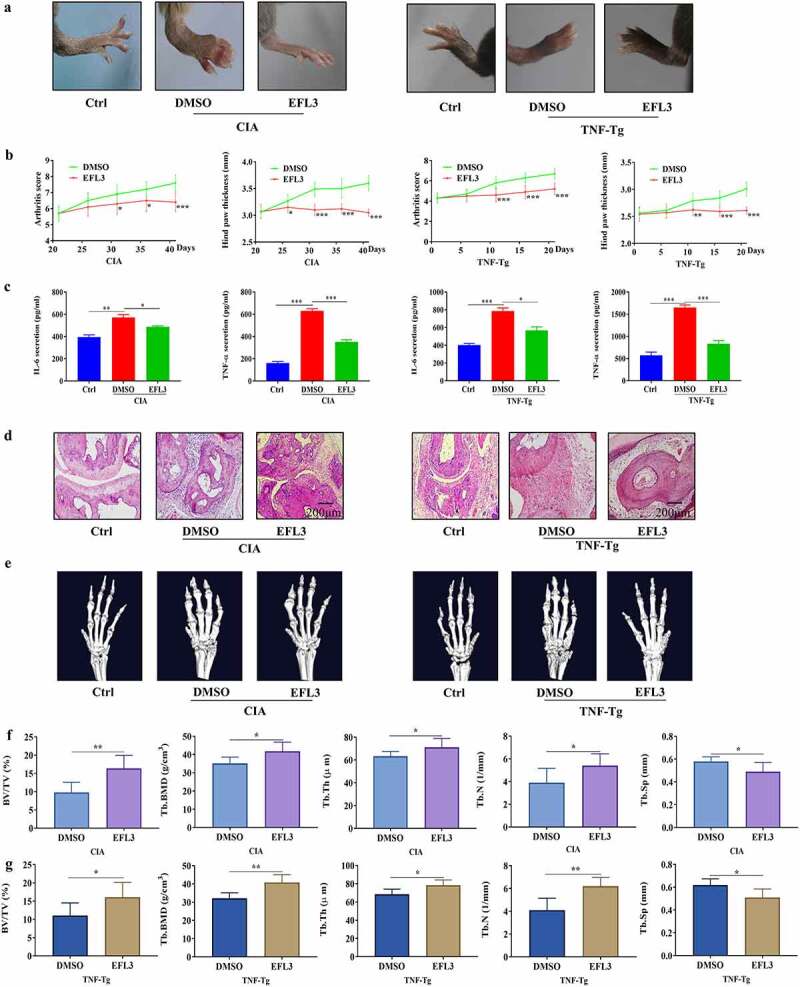
EFL3 inhibits disease progression in CIA models. (a) Photographs of representative hind legs from CIA or TNF-tg mice treated with or without EFL3 (20 mg/kg). (b) Arthritis scores and paw thickness in CIA models were measured every two days after the second immunization. (c) Serum levels of the proinflammatory cytokines TNF-α and IL-1β in the CIA models were assayed by ELISA. (d) Representative histological images of H&E staining of the interphalangeal joints of CIA models. (e) Micro-CT analysis of ankle joints. BMD, BV/TV, Tb. N, Tb. Th, and Tb. Sp of the distal tibia were assessed by micro-CT and 3D reconstruction in CIA (f) and TNF-tg (g) animals. The data are presented as the mean ± SD (*n* = 6). *p < 0.05, ** *p* < 0.01, *** *p* < 0.001.

CIA mice and TNF-tg mice were used to evaluate the therapeutic effects of EFL3 on RA [24]. The data demonstrated that compared with DMSO treatment, intraperitoneal injection of EFL3 (20 mg/kg) prevented arthritic progression, as evidenced by the increases in clinical scores and paw thickness in both CIA and TNF-Tg mice (Figure 3(a,b)). Additionally, the serum levels of TNF-α and IL-6 were increased in the CIA and TNF-Tg mice compared with their parental controls. However, marked reductions in TNF-α and IL-6 in the serum were observed when CIA and TNF-Tg mice were treated with EFL3 (Figure 3(c)).

Histologic analysis demonstrated that upon the development of an arthritic phenotype in CIA and TNF-Tg mice, severe synovial hyperplasia, pannus formation, inflammatory cells infiltration and cartilage and bone damage were observed. However, these pathological characteristics of arthritis were attenuated in EFL3-treated animals (Figure 3(d)). Micro-CT showed that the animals in the EFL3-treated group suffered less bone damage in their ankle joints than the control mice, as evidenced by the increases in trabecular bone mineral density (Tb. BMD), trabecular thickness (Tb. Th), bone volume fraction (BV/TV) and trabecular number (Tb. N) and the decrease in trabecular separation (Tb. Sp) in the distal tibia (Figure 3(e-g)). These results indicated that EFL3 can effectively delay disease progression in arthritis animal models.

### Network pharmacology analysis of EFL3

**Figure 4.**
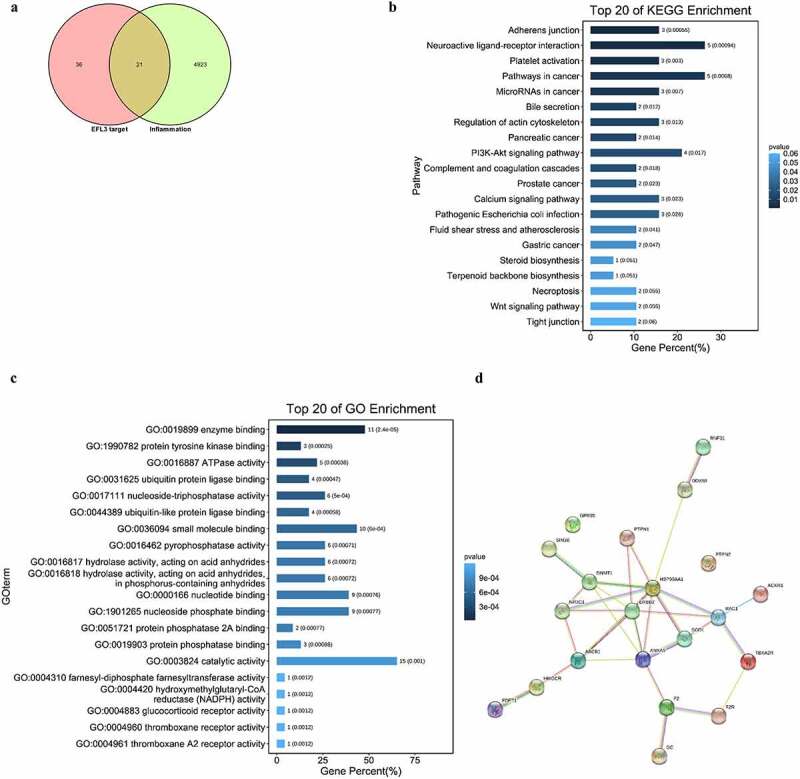
Network pharmacology analysis of EFL3. (a) The intersection of the targets of EFL3 and inflammation. KEGG (b), GO (c) and STRING (d) analyses of the inflammatory targets of EFL3.

To elucidate the molecular mechanism and identify the anti-inflammatory targets of EFL3, network pharmacology analysis was used, which has been widely used to predict targets of traditional Chinese medicine and active ingredients [25]. Importantly, 21 potential targets were predicted (Figure 4(a)). KEGG analysis showed that these targets were significantly enriched in adherens junction and neuroactive ligand–receptor interactions (Figure 4(b)). GO analysis demonstrated that these potential targets were related to enzyme binding, protein tyrosine kinase binding and adenosine triphosphatase (ATPase) activity (Figure 4(c)). In particular, STRING analysis showed that HSP90AA1, ERBB2, RAC1, ANXA5 and DNMT1 were the key nodes (Figure 4(d)).

### Rac1 acts as a novel target of EFL3, and Rac1 inhibition contributes to the antiarthritic effects of EFL3

**Figure 5.**
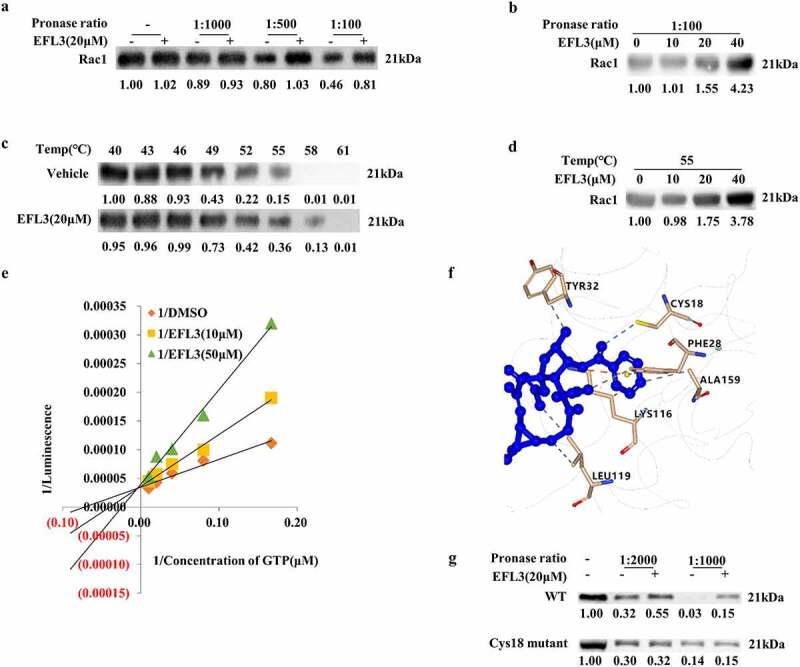
Identifying Rac1 as the target of EFL3. (a) DARTS assays were performed on RA FLSs with EFL3 (20 μM) and proteinase at ratios of 1:1000, 1:500 and 1:100. (b) DARTS assays were conducted on RA FLSs treated with the indicated concentrations of EFL3 and proteinase at a ratio of 1:100. (c) CETSA assays of the stability of the Rac1 protein in RA FLSs at 40°C to 61°C as indicated. (d) The stability of the Rac1 protein in the presence of different concentrations of EFL3 at 55°C. (e) Recombinant human RAC1 protein (20 μM) was pretreated with EFL3 for 30 min, and then the RAC1 GTPase activity was measured. The double reciprocal diagram of GTP concentration and luminescence intensity with EFL3 (10 μM, 50 μM). (f) Molecular docking analysis of EFL3 with Rac1 (PDB ID: 1E96). (g) The DARTS assay was performed on RA FLSs transfected with wild-type RAC1 plasmids and the mutant C18A plasmid. Band intensities were quantified using ImageJ software.

A DARTS assay was then performed, and Rac1 was selected as a potential candidate (Figure 5(a)) due to its close relationship with RA pathogenesis and progression, as well as its effects on a variety of signaling pathways, including NF-κB [26,27]. Then, DARTS samples with a series of protease-to-cell lysate ratios were subjected to Western blot analysis, which showed that azithromycin (AZM) protected the Rac1 protein against degradation (Figure 5(b)). Subsequently, we conducted CETSA to quantify the changes in the Rac1 protein under dynamic experimental conditions [28]. EFL3 increased the resistance of Rac1 to denaturation at 52°C to 58°C compared with DMSO in RA FLSs (Figure 5(c)). Furthermore, EFL3 dose-dependently increased the stability of Rac1 (Figure 5(d)). As the GTPase activity of RAC1 is essential for its own activity [29], the effect of EFL3 on the GTP activity of RAC1 was examined. When incubated with recombinant RAC1 protein, EFL3 inhibited RAC1 GTPase activity in a concentration-dependent manner, and double-reciprocal plots showed that EFL3 might be a competitive inhibitor of RAC1 (Figure 5(e)). To further confirm the binding mode, molecular docking analysis of EFL3 and Rac1 (PDB ID: 1E96) was carried out. As shown in Figure 5(f), EFL3 formed a hydrogen bond interaction with the residues Cys18, a π-cation interaction with the residue Lys166, a π-π stacking with the residue Phe28, and a hydrophobic interaction with residues Ala13, Tyr32, Leu119 and Lys116. These 6 amino acid sites were mutated, and DARTS analysis showed that EFL3 lost its protective effect after Cys18 of Rac1 was substituted with Ala, indicating that Cys18 was the key amino acid required for EFL3 targeting of Rac1 (Figure 5(g)).

**Figure 6.**
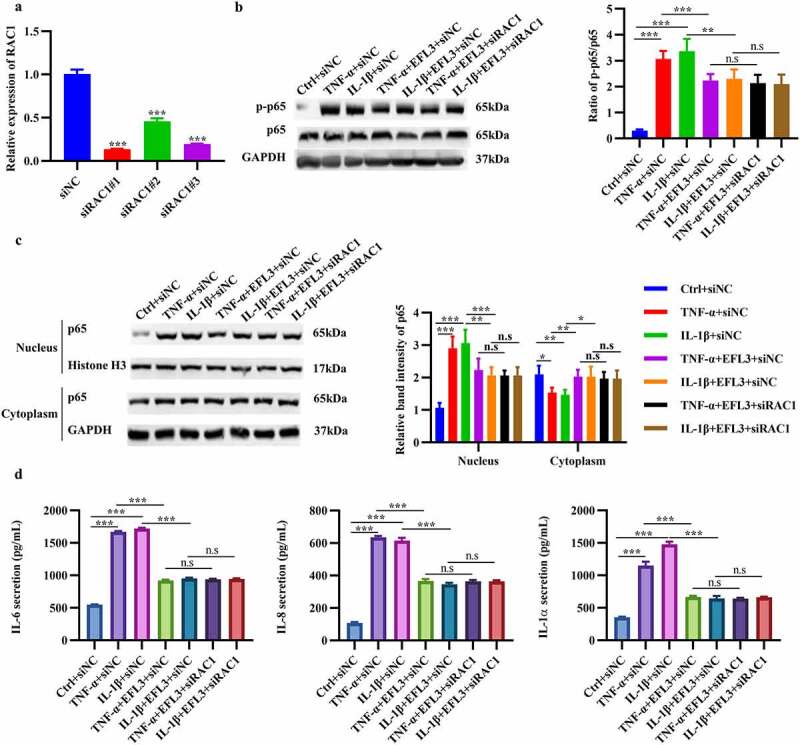
EFL3 inhibits inflammation in RA FLSs via Rac1. (a) The silencing efficiency of the siRNA targeting RAC1 (siRAC1) compared to its negative control siRNA (siNC) was evaluated by RT–qPCR. After exposure to TNF-α or IL-1β for 6 h, RA FLSs with or without RAC1 knockdown were treated with EFL3 or vehicle for an additional 24 h. Then, total protein was extracted to measure the expression of phosphorylated p65 (p-p65) and total p65 (b). (c) Protein extracts from the nucleus and cytoplasm were also subjected to Western blot analysis to measure the expression of p65. (d) IL-6, IL-8 and IL-1α levels in the supernatant of treated RA FLSs were analyzed by ELISA. The data are presented as the mean ± SD (*n* = 3). *p < 0.05, ** *p* < 0.01, *** *p* < 0.001.

To explore whether the antiarthritic effect of EFL3 was achieved by Rac1, Rac1 was silenced in RA FLSs (Figure 6(a)). Rac1 downregulation inhibited the phosphorylation and nuclear translocation of p65 and decreased the production of the inflammatory factors IL-6, TNF-α and IL-1α (Figure 6(b-d)), which was consistent with the effect of EFL3. However, no further decrease in the expression of inflammatory factors was observed when Rac1 was silenced in the presence of EFL3 treatment (Figure 6(b-d)). Furthermore, RA FLSs were transfected with the wild-type RAC1 plasmid and C18A mutant plasmid. The results showed that transfection with wild-type Rac1 could abrogate the anti-inflammatory effects of EFL3 on RA FLSs. However, the Cys18 mutant of Rac1 failed to do so (Figure S1). These results indicate that Rac1 is required for the antiarthritic effects of EFL3.

## Discussion

To date, several genetic and environmental factors associated with RA have been identified; however, the pathogenic mechanism of RA is still incompletely understood [30]. Disease-modifying antirheumatic drugs (DMARDs) and biologics have significantly improved clinical remission [31]. Unfortunately, a notable percentage of RA patients are refractory to the currently available drugs. Therefore, additional treatment strategies and drugs for treating RA should be explored.

FLSs contribute greatly to the maintenance of synovial inflammation and aggressive joint destruction in RA during the pathological progression of RA [23]. In addition, RA FLSs facilitate the differentiation of monocytes to macrophages by secreting chemotactic factors [2]. To date, much attention has been given to identifying novel drugs that target RA FLSs. Previous studies have confirmed the anti-inflammatory activity of EFL3 by inhibiting the production of NO in Abelson murine leukemia virus-induced tumor 264.7 (RAW264.7) macrophages [6]. In this study, we further validated that EFL3 could ameliorate the inflammatory phenotype of RA FLSs by decreasing inflammatory factors and inhibiting the migration and invasion of RA FLSs. CIA models exhibited features similar to those of RA in terms of both histopathological changes and molecular pathogenesis [20]. In addition, TNF-tg animals are develop symmetrical polyarthritis and synovial hyperplasia due to human TNF-α overexpression [32] Importantly, EFL3 could prevent inflammatory progression and joint destruction in CIA and TNF-tg animal models, further supporting its use as a therapeutic drug to treat RA. To further support the therapeutic potential of EFL3, further study is needed to clarify the molecular mechanism by which EFL3 inactivates inflammation in RA.

Previous studies demonstrated that targeting the NF-κB signaling pathway could prevent the progression of inflammation and ameliorate bone erosion in arthritis mouse models [33,34]. Moreover, some clinical drugs, such as iguratimod, show sustained efficacy in RA by inactivating NF-κB [35–37]. Here, we showed that EFL3 could suppress activation of the NF-κB pathway in RA FLSs, further supporting the likelihood that EFL3 could serve as a therapeutic drug to treat RA.

Furthermore, Rho GTPases function as molecular switches and regulate cytoskeletal reorganization during cell migration. Rac1, a Rho GTPase, can promote lamellipodium formation and cell migration partly by regulating actin polymerization [38]. Previous studies reported that Rac1 signaling was closely related to RA and regulates the migration and invasion of RA FLSs [39]. Moreover, RAC1 can activate NF-κB and its target genes, including several proinflammatory cytokines, such as TNF [40]. In this study, Rac1 was identified as a potential target for EFL3, and Rac1-mediated RA FLS activation could be suppressed by treatment with EFL3. In addition, no further inhibition of NF-κB signaling or its target genes was observed when Rac1 was silenced in EFL3-exposed RA FLSs. Taken together, the evidence in this study suggests that Rac1 inactivation is an important mechanism by which EFL3 exerts its antiarthritic effects. These findings suggest that EFL3 could be a promising candidate drug for treating RA.

## Conclusion

Taken together, our in vitro and in vivo experiments have identified a novel anti-inflammatory role of EFL3 in RA FLSs that could prevent disease progression in arthritis animal models. Although previous studies have demonstrated the anti-inflammatory roles of EFL3 in various disease models, its potential remains unclear. The present study also identified RAC1 as the likely target by which EFL3 prevents inflammation and the disease progression of RA. Therefore, EFL3 may serve as a novel treatment agent for RA.

## Future direction of this study

However, this study had several limitations. First, the main merit of herbal ingredients is that they can exert their therapeutic effects through multiple targets. Therefore, in addition to RAC1, further study is needed to verify additional targets of EFL3 to confirm its therapeutic potential in RA. Next, comparative studies should be performed in the near future to compare the efficacy of EFL3 relative to DMARDs and biologics.

## Supplementary Material

Supplemental Material
