# Supplementary material for: Euphorbia factor L3 ameliorates rheumatoid arthritis by suppressing the inflammatory response by targeting Rac family small GTPase 1
Source: Bioengineered. 2022 Apr 27;13(4):10985–98. doi: 10.1080/21655979.2022.2066761 (PMC9208460; doi:10.1080/21655979.2022.2066761)
Supplement: Supplemental Material [file KBIE_A_2066761_SM7522.zip › supplementary/downloadFromZipFile.pdf]

Shandong Medical Biotechnology Center

Approval of Ethical Review of Biomedical Research Involving Animals

NO. SMBC21LL006

Project: The pathological role and mechanism of UPR in Rheumatoid arthritis

Sources of Funding: National Natural Science Foundation of China (NO. 82072850)

Principal Investigator: Wang Lin

Academic Title: Professor Tel: 0531-59567360

Responsible Research Unit: Shandong Medical Biotechnology Center

Research starting and end time: 202101-Now

Review Opinion

After review by the Ethics Committee: the use of experimental animals in this project basically conforms to the "3R" principle, the qualifications of the relevant experiment personnel and the relevant units of the experiment are appropriate, the species, strains, quality grades, and specifications of the animals used in the experiment are basically appropriate, and the research methods are in line with routines. The experimental design basically complies with the relevant animal protection principles, laboratory animal welfare ethics and other ethical requirements. The applicant promises to consciously abide by the relevant animal experiment ethics and accept the supervision and inspection of this committee at any time.

Agree to carry out research on this project.

Ethics Review Committee of Shandong

Medical Biotechnology Research Center

Chairman: ________________
