# Supplementary material for: Euphorbia factor L3 ameliorates rheumatoid arthritis by suppressing the inflammatory response by targeting Rac family small GTPase 1
Source: Bioengineered. 2022 Apr 27;13(4):10985–98. doi: 10.1080/21655979.2022.2066761 (PMC9208460; doi:10.1080/21655979.2022.2066761)
Supplement: Supplemental Material [file KBIE_A_2066761_SM7522.zip › supplementary/Supplementary Table 1.docx]

| Supplementary Table 1 Primers used in this study | | |
| --- | --- | --- |
| Gene | Forward | Reverse |
| IL-6 | CCACCGGGAACGAAAGAGAA | GAGAAGGCAACTGGACCGAA |
| IL-8 | CAGTTTTGCCAAGGAGTGCTAA | AACTTCTCCACAACCCTCTGC |
| IL-1α | ATGGCCAAAGTTCGAGACATG | CTACGCCTGGTTTTCCAGTATCTGAAAGTCAGT |
| MMP-1 | TGTTCTGGGGTGTGGTGTCT | CTGAGCCACATCAGGCACTC |
| MMP3 | CTGGACTCCGACACTCTGGA | CAGGAAAGGTTCTGAAGTGACC |
| GAPDH | GCACCGTCAAGGCTGAGAAC | TGGTGAAGACGCCAGTGGA |
