# Supplementary material for: Euphorbia factor L3 ameliorates rheumatoid arthritis by suppressing the inflammatory response by targeting Rac family small GTPase 1
Source: Bioengineered. 2022 Apr 27;13(4):10985–98. doi: 10.1080/21655979.2022.2066761 (PMC9208460; doi:10.1080/21655979.2022.2066761)
Supplement: Supplemental Material [file KBIE_A_2066761_SM7522.zip › supplementary/western blot images in full size.pptx]

## Slide 1
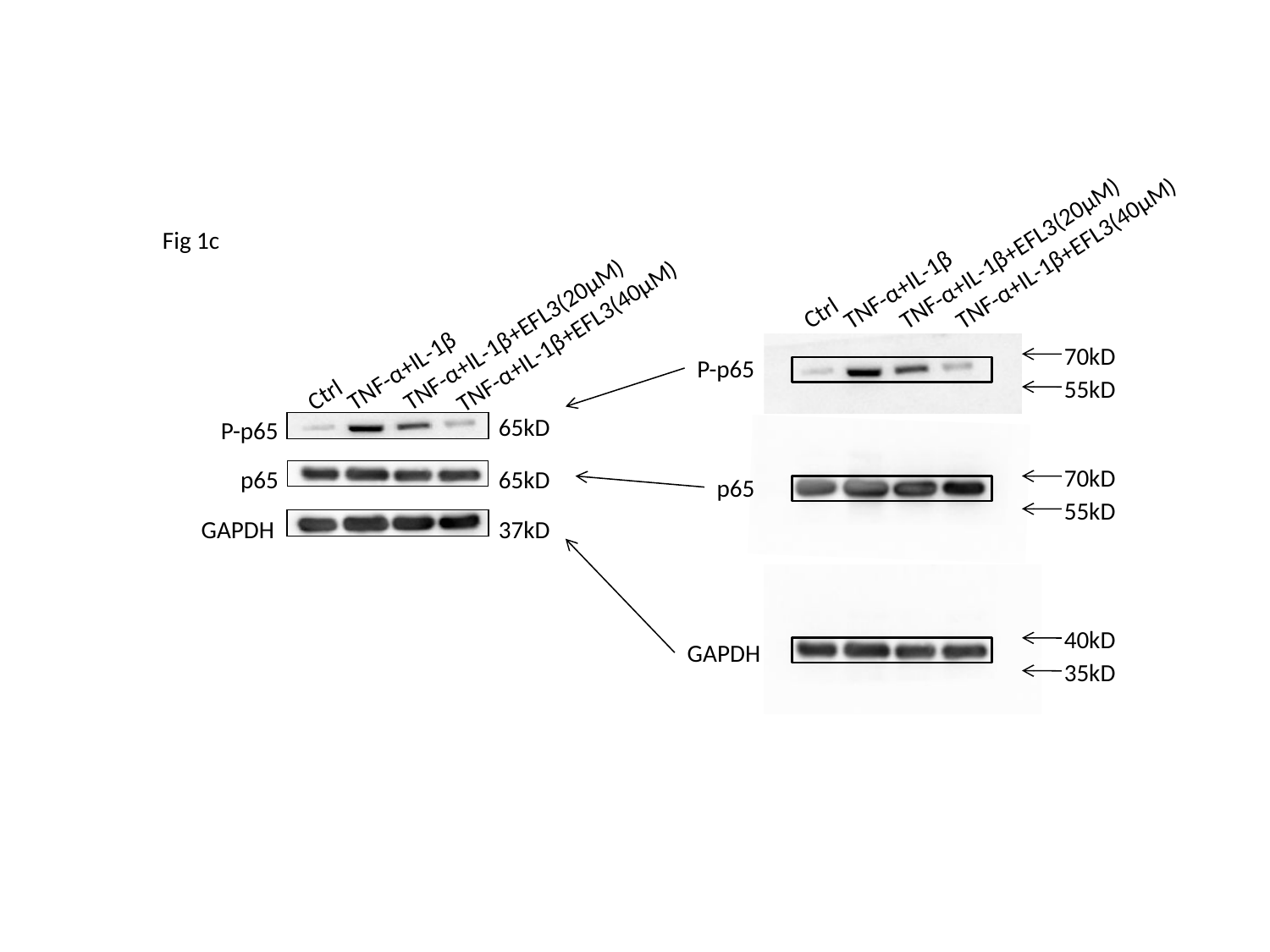

TNF-α+IL-1β+EFL3(40μM)
 Fig 1c
TNF-α+IL-1β+EFL3(20μM)
TNF-α+IL-1β
Ctrl
TNF-α+IL-1β+EFL3(40μM)
TNF-α+IL-1β+EFL3(20μM)
70kD
TNF-α+IL-1β
P-p65
55kD
Ctrl
65kD
P-p65
70kD
p65
65kD
p65
55kD
GAPDH
37kD
40kD
GAPDH
35kD

## Slide 2
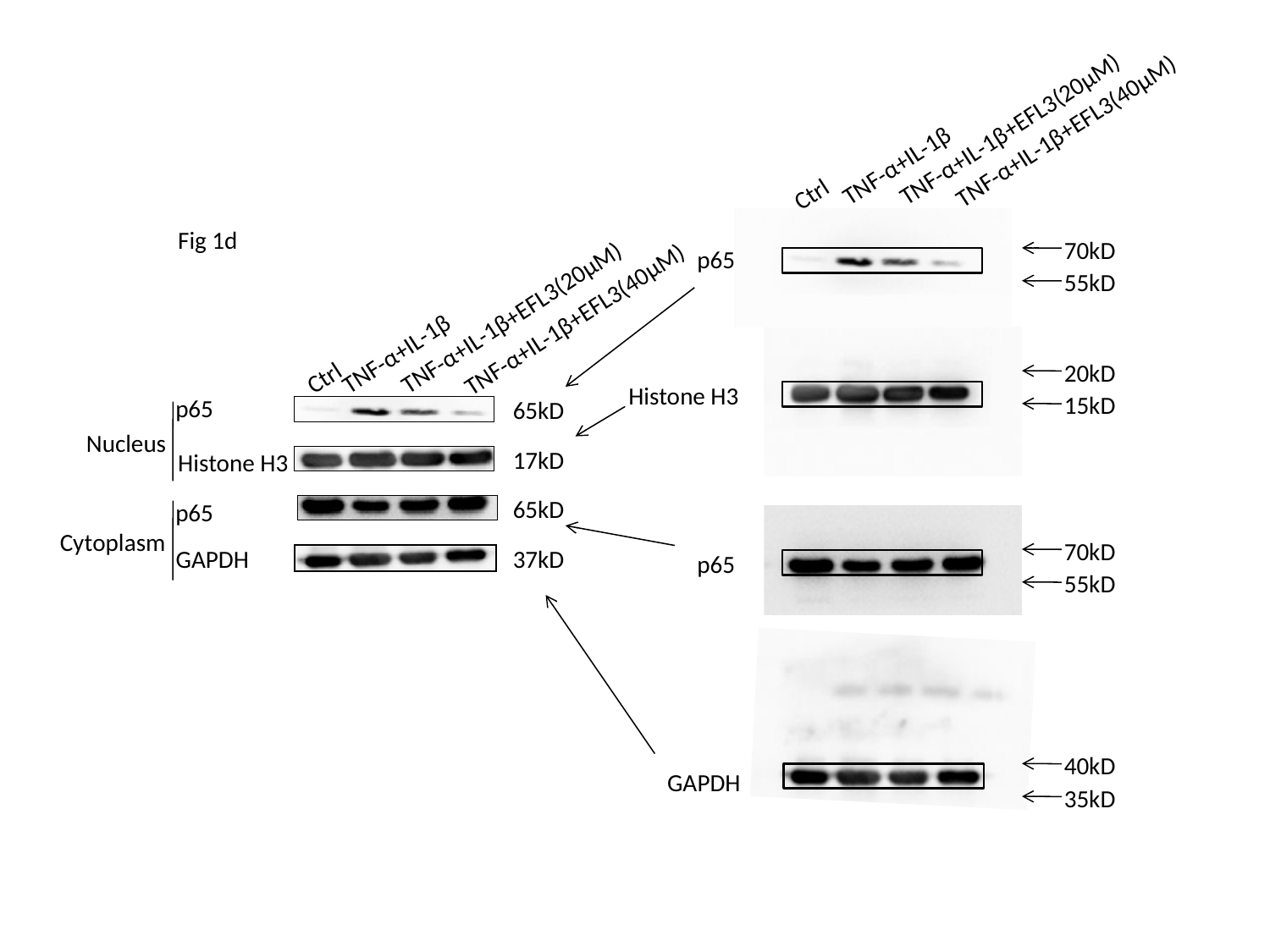

TNF-α+IL-1β+EFL3(40μM)
TNF-α+IL-1β+EFL3(20μM)
TNF-α+IL-1β
Ctrl
Fig 1d
70kD
p65
55kD
TNF-α+IL-1β+EFL3(20μM)
TNF-α+IL-1β+EFL3(40μM)
TNF-α+IL-1β
Ctrl
20kD
Histone H3
15kD
p65
65kD
Nucleus
17kD
Histone H3
65kD
p65
Cytoplasm
70kD
GAPDH
37kD
p65
55kD
40kD
GAPDH
35kD

## Slide 3
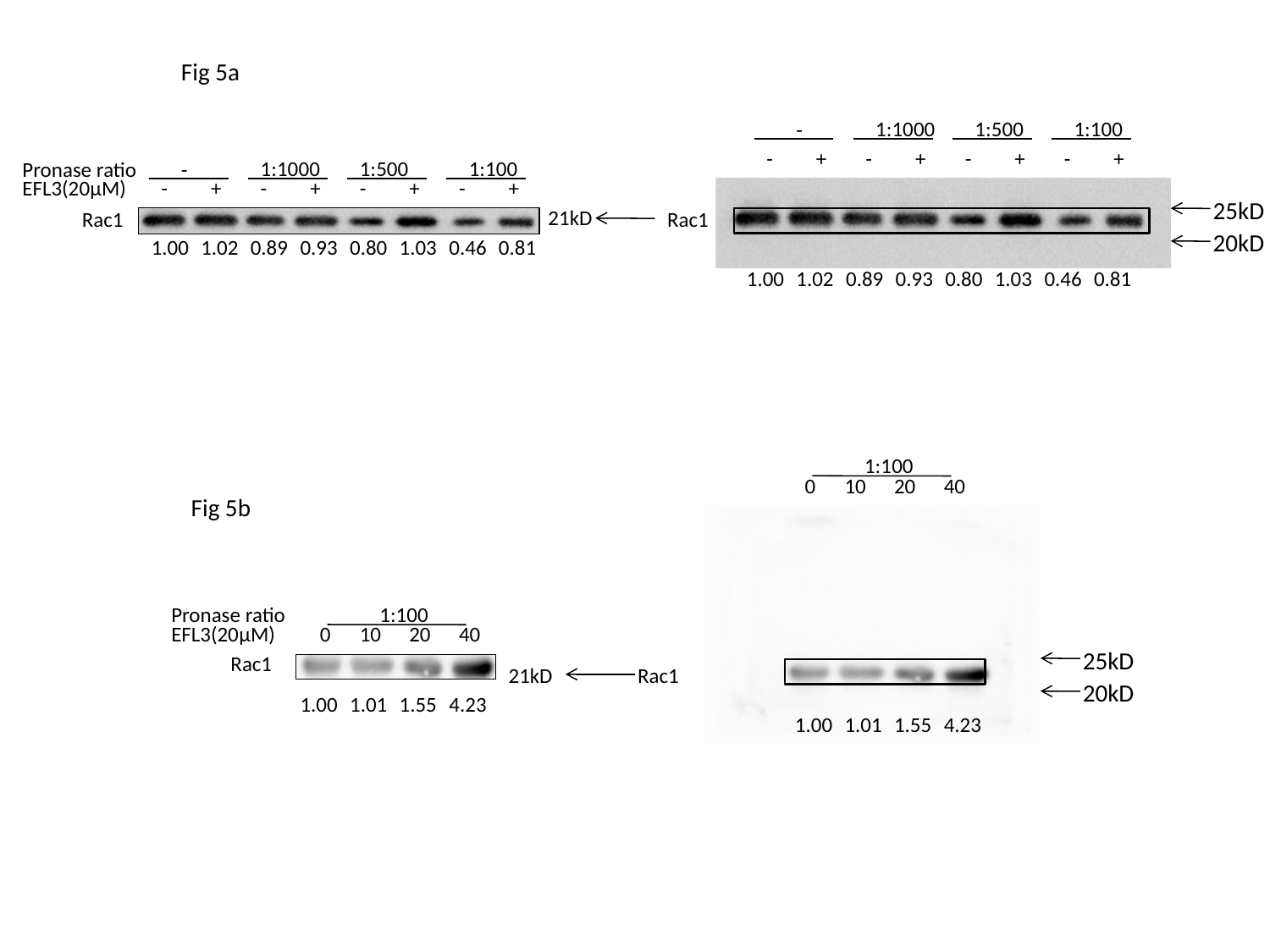

Fig 5a
1:1000
1:500
1:100
-
-
+
-
+
-
+
-
+
1:1000
1:500
1:100
Pronase ratio
-
EFL3(20μM)
-
+
-
+
-
+
-
+
25kD
21kD
Rac1
Rac1
20kD
1.00
1.02
0.89
0.93
0.80
1.03
0.46
0.81
1.00
1.02
0.89
0.93
0.80
1.03
0.46
0.81
1:100
0
10
20
40
Fig 5b
Pronase ratio
1:100
EFL3(20μM)
0
10
20
40
25kD
Rac1
21kD
Rac1
20kD
1.00
1.01
1.55
4.23
1.00
1.01
1.55
4.23

## Slide 4
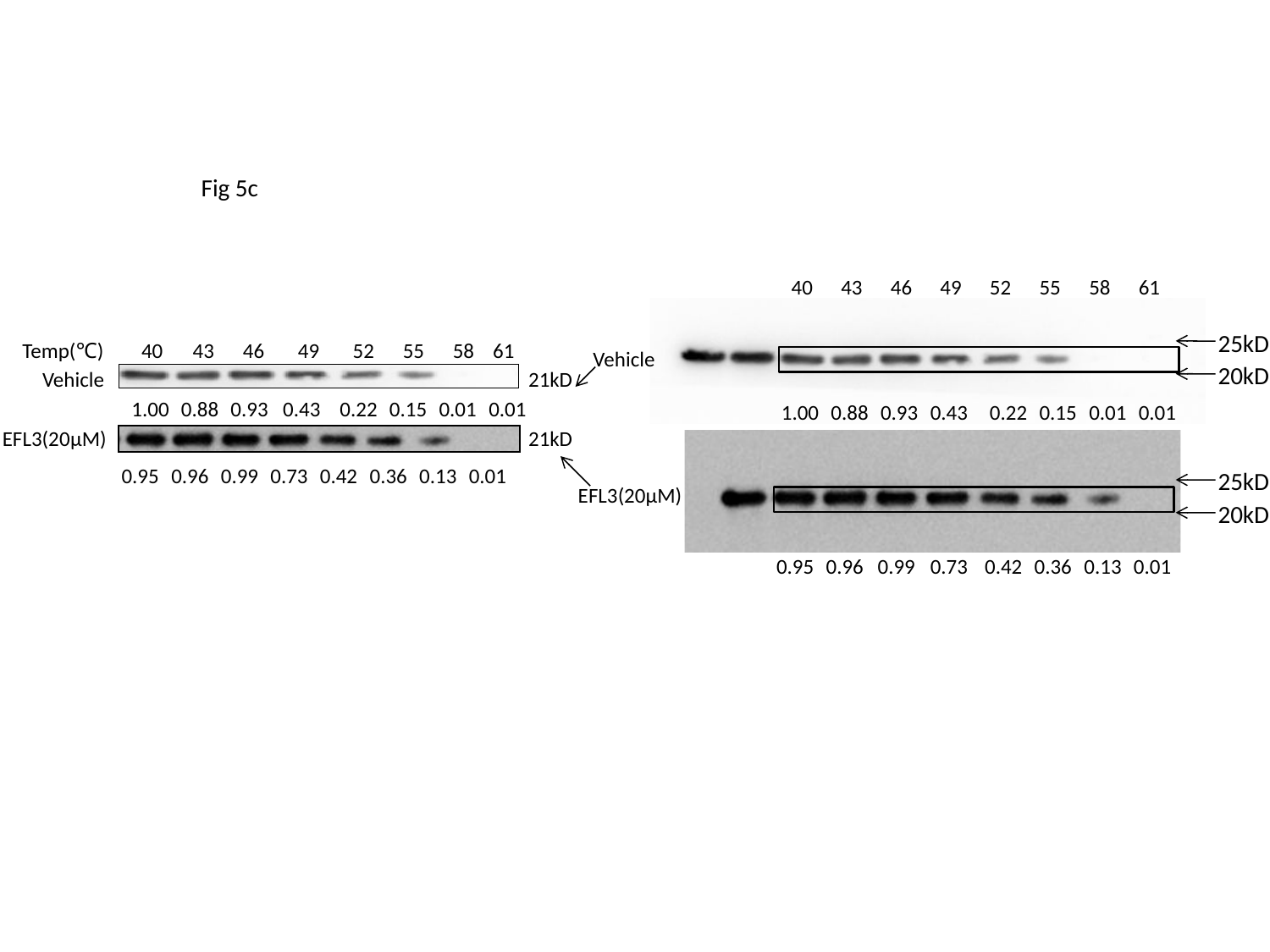

Fig 5c
40
43
46
49
52
55
58
61
25kD
Temp(℃)
40
43
46
49
52
55
58
61
Vehicle
20kD
Vehicle
21kD
1.00
0.88
0.93
0.43
0.22
0.15
0.01
0.01
1.00
0.88
0.93
0.43
0.22
0.15
0.01
0.01
EFL3(20μM)
21kD
0.95
0.96
0.99
0.73
0.42
0.36
0.13
0.01
25kD
EFL3(20μM)
20kD
0.95
0.96
0.99
0.73
0.42
0.36
0.13
0.01

## Slide 5
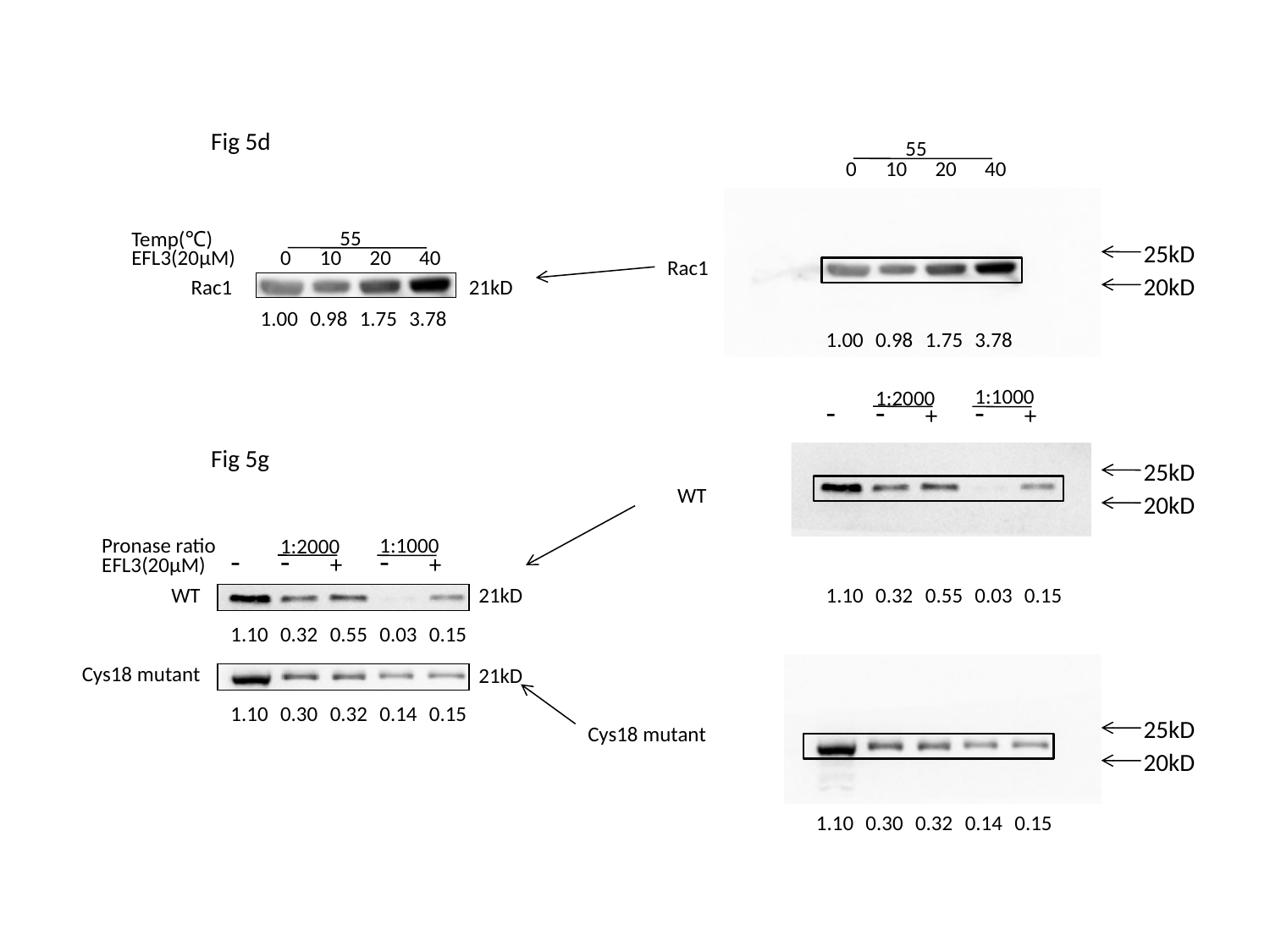

Fig 5d
55
0
10
20
40
55
Temp(℃)
25kD
EFL3(20μM)
0
10
20
40
Rac1
20kD
Rac1
21kD
1.00
0.98
1.75
3.78
1.00
0.98
1.75
3.78
1:1000
1:2000
-
-
-
+
+
Fig 5g
25kD
WT
20kD
Pronase ratio
1:1000
1:2000
-
-
-
+
+
EFL3(20μM)
WT
21kD
1.10
0.32
0.55
0.03
0.15
1.10
0.32
0.55
0.03
0.15
Cys18 mutant
21kD
1.10
0.30
0.32
0.14
0.15
25kD
Cys18 mutant
20kD
1.10
0.30
0.32
0.14
0.15

## Slide 6
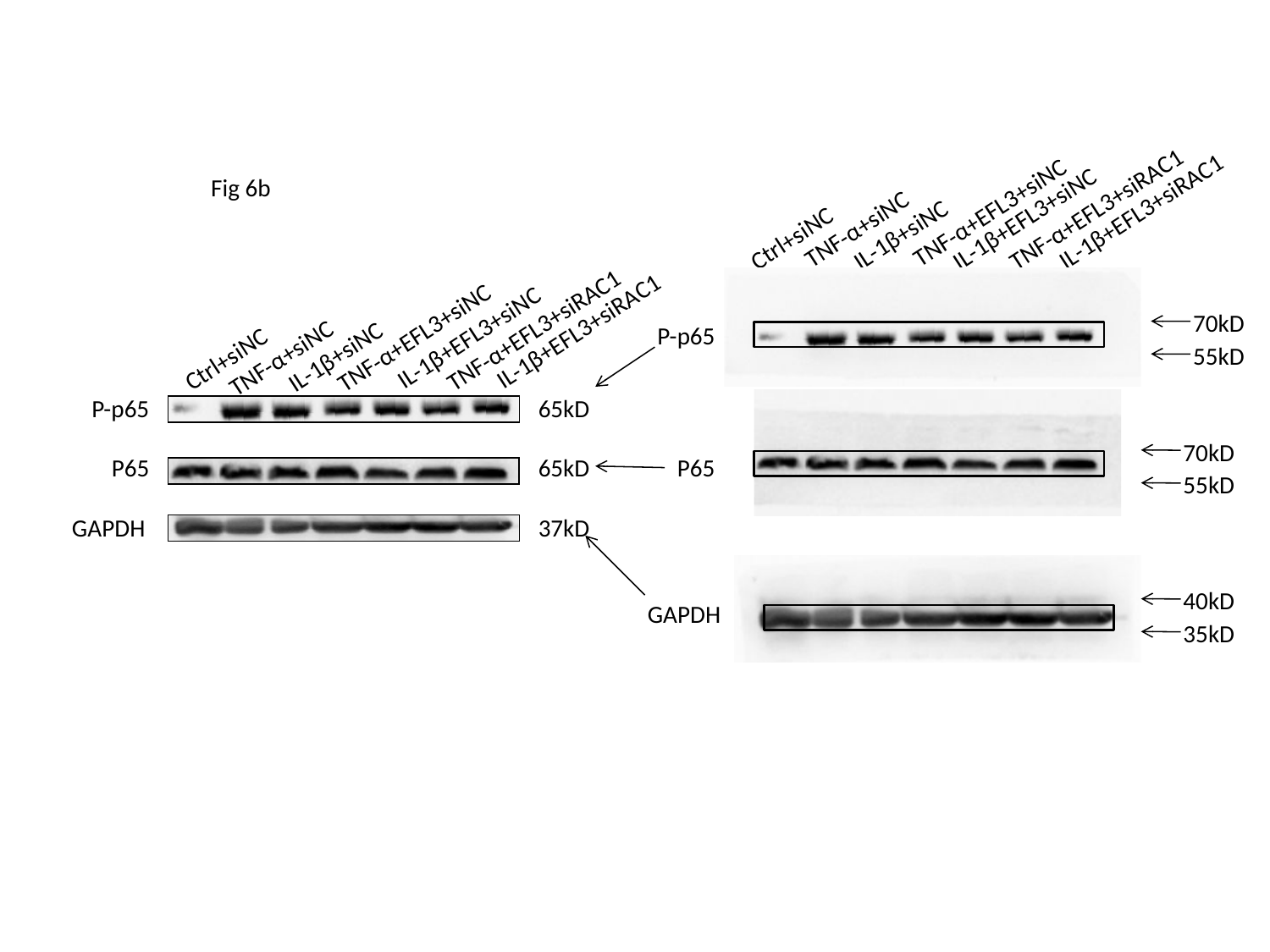

Fig 6b
TNF-α+EFL3+siNC
IL-1β+EFL3+siRAC1
TNF-α+EFL3+siRAC1
IL-1β+EFL3+siNC
IL-1β+siNC
TNF-α+siNC
Ctrl+siNC
TNF-α+EFL3+siNC
IL-1β+EFL3+siRAC1
70kD
TNF-α+EFL3+siRAC1
IL-1β+EFL3+siNC
P-p65
IL-1β+siNC
55kD
TNF-α+siNC
Ctrl+siNC
65kD
P-p65
70kD
P65
P65
65kD
55kD
GAPDH
37kD
40kD
GAPDH
35kD

## Slide 7
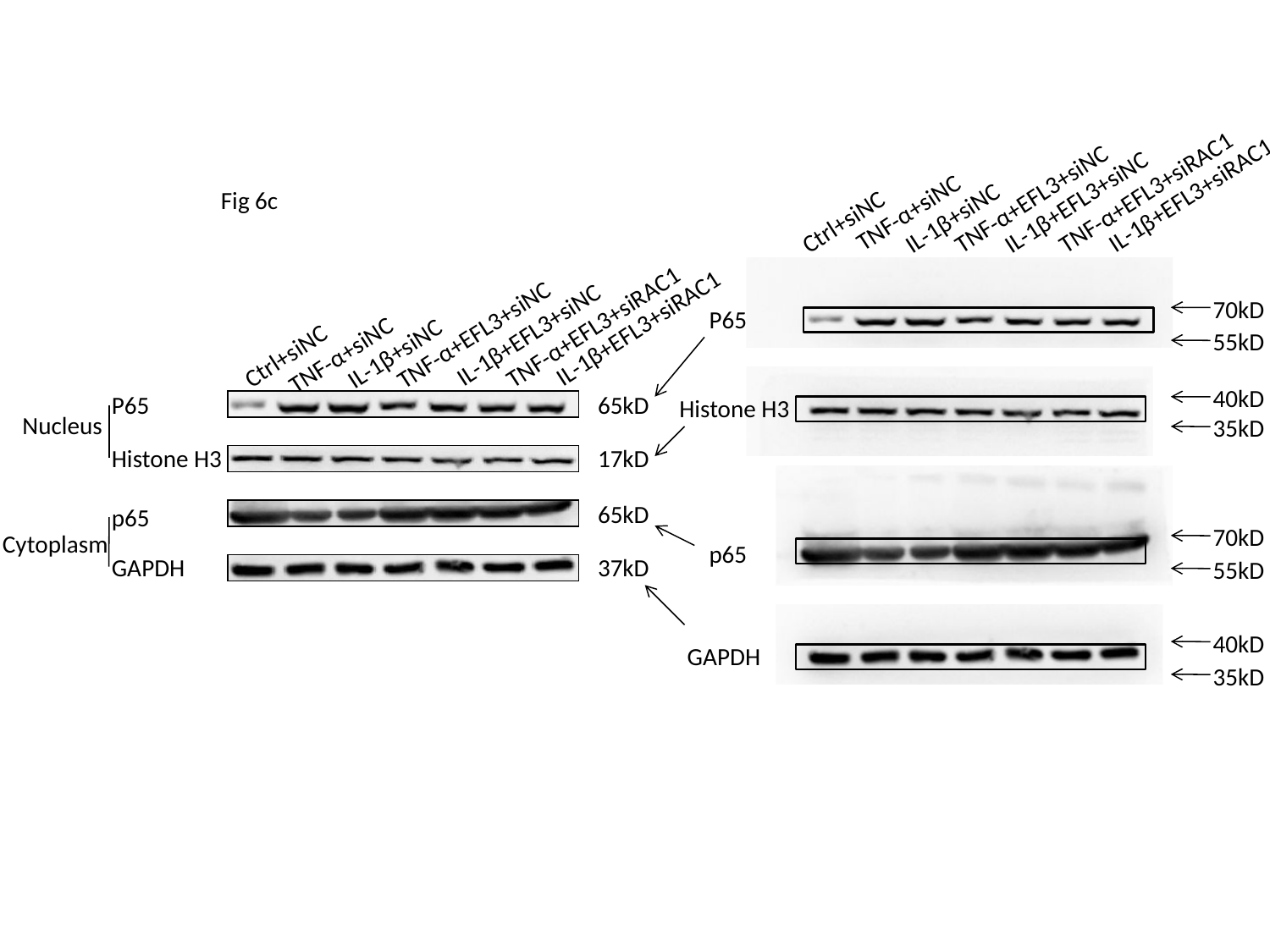

TNF-α+EFL3+siNC
IL-1β+EFL3+siRAC1
TNF-α+EFL3+siRAC1
IL-1β+EFL3+siNC
Fig 6c
IL-1β+siNC
TNF-α+siNC
Ctrl+siNC
70kD
TNF-α+EFL3+siNC
IL-1β+EFL3+siRAC1
P65
TNF-α+EFL3+siRAC1
IL-1β+EFL3+siNC
55kD
IL-1β+siNC
TNF-α+siNC
Ctrl+siNC
40kD
P65
65kD
Histone H3
Nucleus
35kD
Histone H3
17kD
65kD
p65
70kD
Cytoplasm
p65
GAPDH
37kD
55kD
40kD
GAPDH
35kD
